# Etiology of Epizoötic Abortion1Read before the Veterinary Medical Society, University of Pennsylvania.

**Published:** 1898-04

**Authors:** S. J. J. Harger

**Affiliations:** University of Pennsylvania, Veterinary Department, Philadelphia, Pa.


### ETIOLOGY OF EPIZOÖTIC ABORTION.[1]

By S. J. J. Harger, V.M.D.,

UNIVERSITY OF PENNSYLVANIA, VETERINARY DEPARTMENT, PHILADELPHIA, PA.

Unfortunately, the government and the support given to American veterinary schools are such that the bacteriologic investigation of infectious diseases can be made only under considerable disadvantages, and then frequently must their experimental study be made at the personal expense of the investigator. Thus we are frequently obliged, and often with a sense of chagrin, to be de-

[1] Read before the Veterinary Medical Society, University of Pennsylvania.

pendent upon every possible source in order to supply ourselves with the desired information. One of the diseases which may be placed in this category is epizoötic abortion.

This disease is of much interest to the breeder and the farmer. It entails on them much loss, since, when once it has been introduced into a stable, it goes through the entire herd. It has for a long time been generally accepted that the disease is specific—contagious; but as to the identity of the causative agent we possess little or no definite knowledge. As bacteriology is being more generally applied, the causes of contagious diseases become less obscure. Numerous treatises upon the disease in question have from time to time appeared in print, and among the most interesting and conclusive, although, perhaps, not absolutely so, because we are often too much inclined to be enthusiastic over what is new, must be mentioned that of Bang, of Copenhagen.

It has been known for a long time that the introduction into the vagina of healthy pregnant cows of the vaginal contents of cows that have aborted will cause abortion in the former. The Minister of Agriculture of France in 1885 authorized M. Nocard to make a special investigation of epizoötic abortion in cows, which was then very prevalent. Nocard's conclusion indicated that epizoötic abortion was a bacteridian disease of the fœtus and its envelopes. Two different kinds of microbes were found between the uterus and the embryo: a micrococcus disposed in chains of twos and fives, and a short, thick bacillus isolated or associated in pairs. However, the specific rôle of these microbes was not proved, and the inoculation with their cultures of pregnant cows did not induce abortion.[1]

The researches of Bang were more conclusive. A five-year-old cow, served May 21, 1895, was purchased December 19th, and since the 15th of the same month had shown some symptoms of epizoötic abortion, which existed on this farm. She was killed, the genital organs were removed, and an elastic ligature placed around the vagina. The exterior of these organs was normal, the os uteri firmly closed, and the cervix filled with a thick, normal mucus. Between the uterine mucous membrane and the embryo (chorion) was found a straw-colored, mucous, grumous, odorless, abundant exudate of alkaline reaction; in some places it was semi-solid. Placed in a test-tube, this exudate separated into two strata: an upper, serous, cloudy, and yellowish-red; a lower, thick, and yel-

[1] Revue Vet., December, 1897, Etiologie de d'&vortement Epizootique.

lowish-gray. Underneath the chorion was a clear, gelatinous fluid mixed with very fine membranes, due to an œdematous infiltration of the thin connective-tissue layer between the chorion and the allantoid. The allantoid fluid was normal, excepting small clots held in suspension ; amniotic fluid normal, and the umbilical cord œdematous. The fœtus was seven months old, and normal, excepting congestion of the intestines and a small quantity of bloody serum in the peritoneal cavity.

Methylene-blue demonstrated the presence of a large number of small bacteria in the straw-colored exudate, appearing isolated or in dense masses, surrounded by large cells. Most of these organisms in masses were micrococci ; those appearing singly were elongated, oval, and under a high-magnifying power were recognized as small immobile bacilli containing one, two, and three rounded granules ; they stained easily with anilin. A few bacteria were found in the blood of the fœtus, none in the subchorial œdema, and in the intestinal contents many granules that stained, but could not be recognized as bacteria.

From these lesions it would appear that epizoötic abortion is a specific uterine catarrh, caused by a specific bacterium. The uterine exudate, containing epithelial cells, pus, and detritus, is constant, and at the time of abortion, immediately afterward or sometimes before, is discharged from the vagina as a more or less abundant liquid, mucus, purulent, grumous, pale-red, and it can be distingished from that of a metritis by being odorless and appearing immediately after parturition.

Cultures upon serum-gelatin-agar showed in two days in one tube, and in four days in another, small colonies in the form of a zone about 5 mm. from the free surface and 10 to 15 mm. in thickness. These microbes are, therefore, not aërobic, nor yet exactly anaërobic, but rather have a peculiar intermediary position ; the inferior limit of the zone of growth corresponds to the superior limit of a strictly anaërobic bacterium. The colonies are small, round, punctiform, the largest equalling a pinhead. They consist of bacilli similar to those found in the uterine exudate. Upon bouillon, with 5 per cent. glycerin, and upon pure, liquid serum, the cultures were small; upon agar-gelatin, unsuccessful. Cultures supplied with oxygen by means of a solution of pyrogallol remain sterile. Their growth close to the surface of the agar-serum shows that they require oxygen, but in less abundance than is supplied by atmospheric air ; they grow in oxygen in the proportion of 21 per cent. of that contained in the atmospheric air. For other bacteriologic characters I must refer you to the original laboratory work.

Through the kindness of other veterinarians the uterine material, consisting of portions of the afterbirth, removed immediately after delivery, with the hand, as well as the uterine exudate, was procured from twenty-one cases of abortion. In every instance the conditions were identical as to the exudate, which was sometimes mixed with blood, as well as the subchorial œdema ; the chorial vessels were injected, and the cotyledons congested and sometimes hemorrhagic or of a yellowish-gray coloration.

The examination of the exudate in all cases demonstrated the presence of the characteristic bacillus, singly or aggregated in groups. Their number varied, sometimes very numerous, sometimes difficult of detection. Other forms of microbes may enter the uterus from the exterior, but pure cultures of the specific bacillus could always be discovered. In three fœtuses pure cultures, without any trace of foreign microbes, were obtained from the intestinal contents. In one fœtus, five months old, pure cultures were obtained from the blood ; in another the result was negative; the medulla oblongata, the fourth stomach, and the intestines in one case also gave pure cultures of the bacillus. In two cases in which the fœtus was mummified and not expelled, the uterus contained the bacillus, and pure cultures were obtained.

The bacillus possesses great vitality. The uterine exudate, collected in a sterilized flask and placed in an ice-refrigerator for seven months, still gave pure cultures. This may account for the fact that a cow aborting once has a tendency to do so the second time, unless the uterus is thoroughly cleansed.

An English commission (Woodhead, Aitken, McFadyean, and Campbell) proved that the period of incubation is from five to ten weeks, and in four cases pure cultures of the bacillus having been introduced into the vagina, there was no result in nineteen, twenty-nine, thirty-three, and thirty-five days afterward, respectively.

*Experiments* 1 *and* 2.—Two pregnant cows were purchased from an establishment where the disease was unknown: one four years old, and served January 14th ; the other seven years old, and served January 16th. April 14th a considerable quantity of pure bacilli-cultures introduced into the anterior part of the vagina by means of a pipette. After five weeks there was no result, and May 23d and June 4th the injections were repeated. On June 23d signs of abortion appeared in both cows, and the four-year-old aborted the next day; the uterine exudate contained the bacillus. The other subject was destroyed the same day, and bacilli-cultures were obtained from the exudate on the uterine surface. In these cases the period of incubation was ten weeks.

*Experiment* 3.—A cow, pregnant since January 19th, was given a vaginal injection of pure bacilli-cultures. A small calf was born at full term, but it became affected with diarrhœa, and in two weeks had to be destroyed. At the time of parturition the cow discharged a reddish, purulent secretion containing the bacillus. The membranes were œdematous, and a metritis followed. There evidently existed here a uterine catarrh, but the period of incubation was unusually long.

Sheep: In ovine species epizoötic abortion is not as frequent as in cows, but it does exist. It is attributed to a septic infection from the litter, and has been seen on farms where the disease existed in cows. It has been produced by injecting into the vagina the vaginal contents of a cow which had aborted.

*Experiment* 4.—A ewe, pregnant since November 7th, received a vaginal injection of pure cultures January 29th following. After 147 days, a few days before full term, two healthy lambs were born. Upon the chorion and the congested cotyledons was found an exudate which contained the specific bacillus. Parturition was a little premature, and the period of incubation was sixty-four days.

*Experiment* 5.—A second ewe, pregnant since November 11th, was treated in the same manner, and was destroyed April 3d. The uterus contained two lambs; the embryo was covered with a small quantity of exudate not containing the bacillus. The bacillus introduced into the vagina, therefore does not always enter the uterus.

*Experiment* 6.—Eighteen cc. of a culture were injected into the jugular vein of a pregnant ewe. The next day the symptoms were fever, difficult respiration, and loss of appetite. Twelve days afterward were born two small lambs which developed normally. The fœtal membranes were œdematous, and showed the presence of the bacillus in abundance. The bacillus can, therefore, enter the uterus through the circulation.

*Experiment* 7.—A pregnant ewe, having a wound on a posterior member, received 8 c.c. of culture into the jugular vein, and on the seventh day threw a lamb of almost four pounds. The only symptom was a rise of temperature. Three days after parturition the female died from pleurisy and peritonitis. The membranes showed the specific bacillus.

*Experiment* 8.—A mare, eight years old and in foal, received an injection of the cultures of the bacillus into the jugular vein. The only consecutive symptom was a rise of temperature. She foaled in twenty-eight days; the foal died the following day from atalectasis—non-inflation of the lungs. The uterine exudate contained the specific bacillus and reproduced cultures.

These experiments aim to prove that epizoötic abortion in the mare is due to the same cause as that in cows, and that there is a probability of contagion between the disease in the cow and the ewe. It is said that goats have aborted before term in stables in which cows were suffering from abortion at the time.

The microbe may enter the uterus through the circulatory system or by the genital passages; nothing is known upon this point as to the respiratory and digestive tracts. The infection of the female may take place in the field in which abortion has occurred.

A cow which has aborted has a tendency to do so again the following two years; but each year this tendency diminishes, and unless new cows are introduced into a herd the disease has a tendency to exterminate itself. Each year the abortion takes place later during gestation. Sometimes the following year the animal remains pregnant. The animal seems to acquire immunity in a few years. Primipara are those most frequently affected.

Again, we must not lose sight of the bull in the transmission of the disease. It may be transmitted by means of a specific urethritis in the same manner as gonorrhœa. Instances have been known where the introduction of a bull, or serving the cows by the bull of a neighboring farm, has been closely followed by an outbreak of this disease.

The prophylactic treatment consists in isolation, the usual antisepsis of the genital passages, and disinfection of the stable. The carbolic-acid treatment seems to have given excellent results exclusive of the fact that the disease tends to exterminate itself in time. Ten c.c. of a 2-per cent. solution of carbolic acid are given hypodermically every two weeks until five doses are given. This treatment has immediately arrested the disease, not one animal aborting afterward.

Another treatment which now suggests itself is the use of antitoxin of epizoötic abortion, the healthy animals being injected as soon as the presence of the disease is recognized.

